# Pedigree-Based Genetic Diversity in the South African Boerboel Dog Breed

**DOI:** 10.3390/ani14060975

**Published:** 2024-03-21

**Authors:** Ripfumelo Success Mabunda, Khathutshelo Agree Nephawe, Bohani Mtileni, Mahlako Linah Makgahlela

**Affiliations:** 1 Department of Animal Sciences, Tshwane University of Technology, Private Bag X680, Pretoria 0001, South Africa; ripfumelomabunda94@gmail.com (R.S.M.); nephaweka@tut.ac.za (K.A.N.); mtilenib@tut.ac.za (B.M.); 2 Agricultural Research Council, Animal Production, Private Bag X2, Irene 0062, South Africa; 3 Department of Animal, Wildlife and Grassland Sciences, University of the Free State, P.O. Box 339, Bloemfontein 9301, South Africa

**Keywords:** genetic contribution, genetic diversity, inbreeding, influential ancestors

## Abstract

**Simple Summary:**

Several pedigree-based analyses of dog breed populations have revealed increased levels of inbreeding worldwide. These inbreeding levels threaten dogs’ fitness and adaptability, population development, and survival, as well as breeds’ economic importance and breeding goals. Therefore, determining the population structure of the Boerboel dog breed is critical for monitoring and controlling the loss of genetic diversity through optimal breeding programs in order to preserve its unique genes. This study’s findings revealed that the Boerboel dog breed has less genetic diversity, implying increased homogeneity, and there is unequal founder contribution to the current population’s genetic variation. Future breeding strategies would need to avoid the mating of closely related animals and the use of popular males.

**Abstract:**

The Boerboel dog breed (BBD) is indigenous to South Africa (SA) and plays an important role in safeguarding homes and farms. The Department of Agriculture, Land Reform, and Rural Development (DALRRD) classifies the BBD as a protected species, and it is valued for its intelligence, boldness, and strength, as well as for continually ensuring the safety of its owners. The aim of this study was to investigate genetic diversity within the BBD population using pedigree information. The original BBD data, which contained 87,808 records, were obtained from the Integrated Registration and Genetic Information System (INTERGIS). After editing, the pedigree data included 87,755 records of animals born between 1971 and 2019. Pedigree analyses were performed using PEDIG (Fortran 77 software) to determine the completeness, inbreeding coefficients, and genetic diversity as defined by the genetic contributions of the most important ancestors of the current animals. This study identified 91.2% inbred animals in the BBD population, with an average and maximum inbreeding of 7.5% and 50% of inbred animals, respectively. The estimated inbreeding rate per year was 0.20% with an effective population size of 83.1. The most influential ancestors explained 82.63% and 80.92% of the total genetic variation for males and females in the studied populations, respectively. Only 10 important ancestors explained more than 50% of the entire population’s genetic diversity. The numbers of founders (f) were 348 and 356, and the effective numbers of founders ($f_{e}$) were 57.4 and 60.1, respectively, for males and females. The numbers of founders were higher than the effective numbers of founders, implying a loss of genetic diversity due to unequal founder contributions. The BBD population was not critically endangered based on the inbreeding rates and effective population size; however, the population experienced a significant loss of genetic variability, unequal genetic contributions by founders, and a genetic bottleneck. Future breeding strategies could benefit from using equal proportions of parent stock and including new genetically distant breeds.

## 1. Introduction

The Boerboel dog breed (BBD), which is also known as the ‘Boer mastiff’, is a large dog breed created over the last hundred years in Southern Africa for safeguarding families [1]. It is a guard dog developed from various African and European breeds such as the Bulldog, Bullmastiff, and Rhodesian Ridgeback. Purebred Bullmastiff dogs were imported from Britain by De Beers in 1938 to serve as guard dogs in South African diamond mines at the time; the phenotypic characteristics of these animals are certainly included in the BBD as we know it today [2]. The development of the BBD was promoted by the South African Boerboel Breeders’ Association (SABBA), the union that began the rehabilitation of the breed from mongrel outcasts to “purebred” in 1983 in order to have a South African dog breed [3]. It is indigenous to South Africa, as defined by the DALRRD’s Animal Improvement Act (AIA) 62 of 1998. The South African Boerboel Breeders’ Society (SABBS) has been designated as the breed’s custodian under the AIA (62 of 1998), ensuring the preservation of its genetic diversity and long-term gene conservation.

The BBD is a manageable, reliable, obedient, intelligent, and fearless dog with a strong protective instinct and loyalty to owners. Furthermore, the breed was admired for its bravery, endurance, and ability to hunt leopards and baboons, as well as for its constant vigilance and constant contribution to the safety of its owners. According to the SABBS standard, the BBD is a large dog with a strong bone structure and muscularity [4]. Common coat colors of the breed include brown, brindle, black, fawn, reddish-brown, and various other shades of brown. The recent appearance of black-colored dogs within the BBD population created a conflict between the SABBS recognizing the dogs and the Kennel Union of South Africa (KUSA) not accepting these as purebred [4,5].

The South African Stud Book, which is the registering authority of several breeds and species, declared the BBD to be threatened, which is possibly due to its high inbreeding levels, potentially affecting the breed’s adaptability and survival and increasing the risk of extinction [6]. Concerns about the effect of the decreased genetic diversity on health and performance have enabled the development of improved genetic management practices [7]. Because pedigree dogs are kept in small, closed groups without external gene flow and very few dogs are used for breeding [8], inbreeding consequently affects their performance, reproductive qualities, and adaptability [9]. To avoid high levels of inbreeding, inbreeding depression, loss of genetic diversity, and severe genetic drift, small populations require monitoring and control of genetic variation [10]. Characterization of population structure within breeds is required for assessing a dog breed’s future reproductive potential [11] and monitoring gene flow by providing information on the population’s founding ancestors and their contributions to the existing population’s variability [12]. Furthermore, it is necessary to ensure that animals with high levels of inbreeding (>6.25%) are excluded in mating systems [10]. This can prevent the propagation of harmful recessive genes, genetic diseases such as hip and elbow dysplasia [13,14], and phenotypic defects that can reduce fitness and adaptation to offspring [15]. One goal of genetic conservation is to preserve as much genetic diversity as possible in the founder population [16] to control the effects of genetic drift and keep inbreeding to a minimum [17]. Fortunately, many breeds have pedigree data, which have been used to monitor and assess genetic diversity [18,19]. However, pedigree information is limited by the start of recording and missing or incorrect recordings [20]. Actual animal genotypes are actively exploited in conservation studies to accurately estimate genetic diversity and inbreeding [8,21,22]. Genomic data enable accurate assessment of the population structure based on actual versus expected genetic diversity [23]. The availability of animal genome information, on the other hand, is still limited by the cost of genotyping.

Limited research exists on the genetic diversity of any measured type for the South African BBD population. Knowledge of the BBD’s population structure would be a necessary step towards designing sustainable conservation and improvement strategies. The objective of this study was to investigate the genetic diversity within the BBD population using pedigree information.

## 2. Materials and Methods

### 2.1. Pedigree Data

Pedigree information of the BBD for the period 1971 to 2019 was obtained from the Integrated Registration and Genetic Information System (INTERGIS). These included 87,808 animal records, constituting 43,682 males and 44,084 females, as well as 42 animals without sex category records. The pedigree contained information such as animal identification, sire, dam, birth date, sex, birth status, breeder identification, service code, registration number, and name of the breed. The original data were edited to ensure that parents appeared before offspring and that there were no duplications, and they were also organized to remain with animal identification, sire, dam, birth date, sex, and breeder identification as required by the software used for analysis. The edited data encompassed 87,755 records (42,030 males and 42,331 females), including founders (animals with unknown parents).

### 2.2. Pedigree Analysis

#### 2.2.1. Pedigree Completeness

The PEDIG software of Boichard [24] with various sub-programs that operated independently was used to estimate the genetic diversity parameters. The sub-program *ngen.f* was used to evaluate the quality and depth of the pedigree, as well as the completeness of the pedigree as measured by equivalent complete generations (EqGs), which measured the percentage of known ancestors (parents, grandparents, and great-grandparents) from all generations for each generation. The formula from Boichard et al. [25] was used to determine the pedigree completeness:

$$\frac{1}{N\;}\sum_{j=1}^{N}\sum_{i=1}^{nj}\frac{1}{2gij}$$

where N is the number of animals in the reference population (defined as animals born between 1971 and 2019), nj is the total number of ancestors of animal j, and gij is the number of generations between animal j and its ancestor i [26].

#### 2.2.2. Estimation of the Inbreeding Coefficient

The edited pedigree with 87,755 records was used to estimate the inbreeding coefficients (F). The sub-program *meuw.f* from the method proposed by Meuwissen and Luo [27] was used to calculate individual values of F in the population. The inbreeding coefficient was calculated as

$$F=\sum [({1/2)}^{n}*\left({1+F}_{A}\right)]$$

where F represents the inbreeding coefficient, n is the number of individuals in the inbreeding path, and $F_{A}$ represents the inbreeding coefficient shared by the relative and the inbred individual’s parents. We further calculated the rate of inbreeding, and the effective population size (Ne) was determined using the following formula:

Ne=1/2∆F

#### 2.2.3. Estimation of the Probabilities of Gene Origin

Probabilities of gene origin refer to the probability that a gene originated from any of its founders (grandparents, great grandparents, and great-great grandparents) [25]. The computation requires a defined reference population (RP), which is a set of animals of a particular sex that were born in a defined period to trace back to their ancestors. In this study, the RP was defined as animals born between 2009 and 2019, which included 36,546 animals (18,196 males and 18,350 females), and analyses for males and females were performed separately. The sub-program *prob_orig.f* from the method developed by Boichard et al. [25] was used to trace back to the most important ancestors that contributed to the genetic diversity of the aforementioned animals in the RP. The genetic diversity was defined as the marginal genetic contribution of the important ancestor that was not explained by other ancestors while looking at the probabilities of gene origin. The estimated cumulative genetic contribution represents the total genetic variation explained by the population’s most important ancestors. The marginal genetic contributions were calculated using the formula from Boichard et al. [25] as follows:

$$p_{k}=q_{k}\left(1-\sum_{i-1}^{n-1}a_{i}\right)$$

where $p_{k}$ is the marginal genetic contribution of the k ancestor that is not yet explained by the n−1 ancestors that have already been selected, $q_{k}$ is the expected genetic contribution of founder k to the genetic diversity of the population, and $a_{i}$ is the number of known ancestors in a generation.

In addition, other measures of diversity estimated using *prob_orig.f* included the total number of founders (f), which was defined as the number of animals with unknown parents [28], and the effective number of founders ($f_{e}$), which was the number of founder animals with the same contribution to the production of genetic diversity in the existing population [25]. This was calculated as follows:

$$f_{e}=1/\sum_{k=1}^{f}q_{k}^{2}$$

where $f_{e}$ represents the effective number of founders, f is the number of founders in the population, qk is the expected contribution of founder k to the genetic diversity of the population, and the effective number of ancestors ($f_{a}$) is defined as the small number of ancestors that are important in controlling the complete genetic diversity and represents the bottleneck in the population. The ($f_{a}$) values were determined from the genetic contributions of ancestors with non-zero marginal genetic contributions as follows:

$$f_{a}=1/\sum_{k=1}^{f}p_{k}^{2}$$

where $f_{a}$ is the effective number of ancestors, f is the number of founders in the population, and $p_{k}$ is the marginal genetic contribution of ancestor k that is not yet explained by the n−1 ancestors that have already been selected.

## 3. Results

### 3.1. Pedigree Depth and Completeness

**Figure 1:**
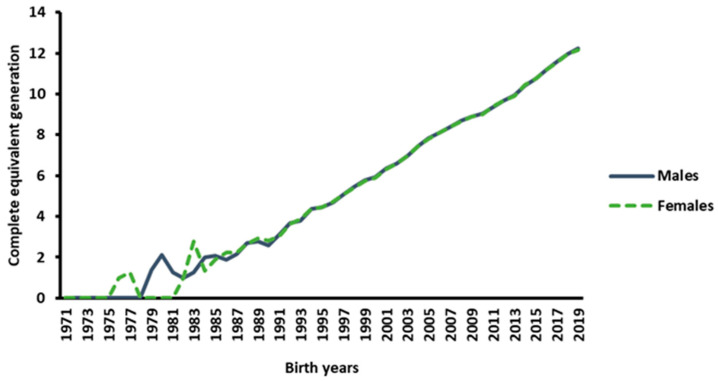
Pedigree completeness as determined with the EqGs for the years 1971 to 2019.

The pedigree completeness as determined with the EqGs is shown in Figure 1 for the period 1971 to 2019. Pedigree recording was initiated in 1971, and an uneven number of founding animals of the current population was shown. As the pedigree recording continued, the pedigree depth increased over time, reaching an average of 12.2 generations for animals born in 2019. For males, the number of generations increased from 1.38 in 1979 to 12.23 in 2019, and for females, it increased from 1.0 in 1976 to 12.17 in 2019.

### 3.2. Estimated Inbreeding Coefficients

Inbred animals (non-zero inbreeding coefficients) made up 80,014 (91.2%) of the 87,755 animals in the pedigree, which was an indication of decreased genetic diversity. Table 1 shows the inbreeding classes of the inbred animals in the pedigree. The population’s lowest percentage of inbred animals (35.8%) had inbreeding coefficients between 0 and 5%, while 64.02% of the animals had inbreeding coefficients above 5%. The average inbreeding of inbred animals was 7.5%, with a maximum of 50%, indicating high inbreeding in the population.

**Figure 2:**
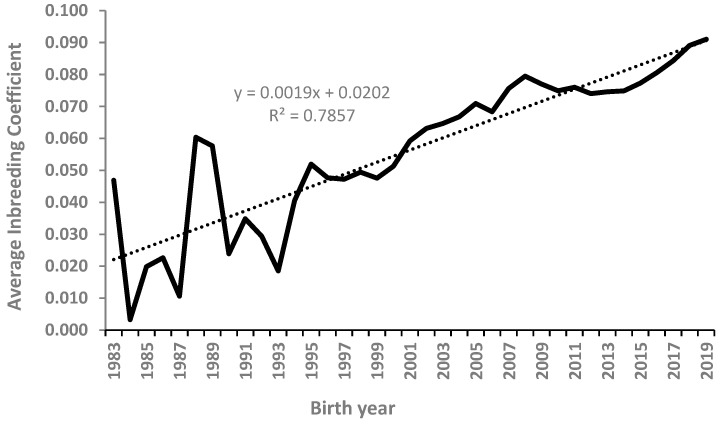
Average inbreeding coefficient by year of birth in the Boerboel dog breed.

Figure 2 presents the average inbreeding coefficient by year of birth in the Boerboel dog breed. Inbreeding increased steadily from 0.047 to 0.091 over the period from 1983 to 2019, respectively. The rate of increase per year, 0.20%, fell at the borderline for ensuring sufficient genetic variability [29]. The observed rate of inbreeding was lower than that reported for the South African Boxer breed (0.42%) over the period from 1960 to 1980 [30]. The rates of increase in inbreeding in both South African dog breeds were, however, lower than the critical rate of increase of 0.5% [29,31].

### 3.3. Estimated Probabilities of Gene Origin

**Figure 3:**
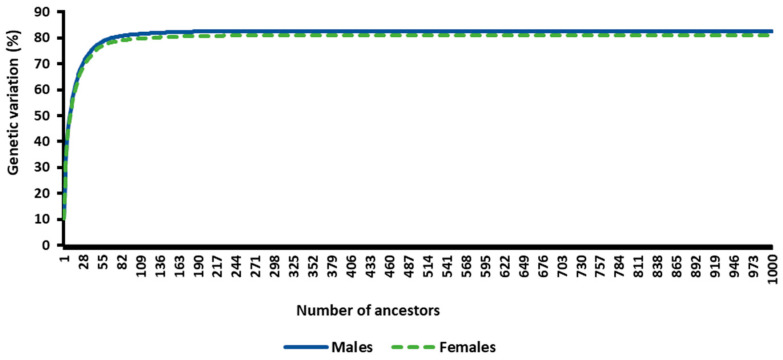
The cumulative genetic variation explained by the reference population’s most important ancestors (birth years: 2009–2019) for males and females.

The total genetic variation as defined by the cumulative genetic contributions from the most important ancestors of the RP (2009–2019) was 82.63% for males and 80.92% for females. Only 10 ancestors explained 50% of the population’s genetic diversity when the RP was defined separately for males and females compared to the additional 32.63% and 30.92% explained by the remaining 990 most important ancestors. Figure 3 presents the cumulative genetic variation explained by the RP’s most important ancestors (birth years: 2009–2019) for males and females.

#### Estimated Measures of Genetic Diversity

The estimated measures of genetic diversity are summarized in Table 2. The numbers of founders observed for males and females were 348 and 356, respectively, and the $f_{e}$ values were 57.4 and 60.1, respectively. The $f_{a}$ values of males (27.93) and females (29.5) were lower than the values of $f_{e}$. The $f_{e}$*/*f ratios were 0.16 and 0.17, indicating low values, whereas the $f_{e}$*/*$f_{a}$ ratios were 2.1 and 2.0, indicating high values for males and females, respectively. The effective population size (Ne), which measured the amount of genetic variability within a population and was calculated from the individual inbreeding rate at 83.1, was within the recommended range of sizes [32,33].

## 4. Discussion

The objective of this study was to investigate the level of genetic diversity in the BBD population using pedigree information. In the studied pedigree of the BBD, both parents were known for 96.1% of the animals, and only 3.9% of the animals had parents that were both unknown, indicating that the pedigree information used for the analysis was sufficient [11]. Improvements over time in pedigree recording processes and the availability of computerized animal recording systems resulted in the reliability of the studied pedigree data. This was ascertained by the average EqGs of 12.2, where the EqGs indicates the depth and quality of pedigree data [34]. The completeness of pedigree information ensures reliability in the estimation of population parameters such as inbreeding [25]; missing 10% of the pedigree information is enough to underestimate inbreeding and, consequently, result in poor monitoring strategies [28]. A pedigree with similar EqGs levels was used by Mäki [8] for Nova Scotia, and a slightly deeper pedigree was used for the Czech Spotted Dog by Machová et al. [35]. Continued efforts are needed for the BBD to maintain the quality of pedigree recording and continuously enhance the pedigree depth.

The BBD population is a closed population with very high inbreeding levels that are exceeding the Food and Agriculture Organization’s (FAO’s) recommended limit of 5% [36]. This indicates a greater loss of diversity, which requires attention to ensure the sustainability of the dog breed. Inbred animals are produced through mating between first cousins and have inbreeding coefficients larger than 6.25% [37]. The average inbreeding coefficient of inbred animals was 7.5%, which was higher than that of first-cousin mating. This could indicate that mating was performed between closely related individuals on purpose—at least between animals with the same coat color—for a specific temperament or because of changing breed standards and market requirements [38,39,40]. All of these factors can affect the long-term selection response and genetic diversity, and they increase the risk of inbreeding depression [41,42]. The inbreeding levels in this study were similar to those previously reported in SA for the BBD breed [6] and elsewhere for the Tatra Shepherd [43], Bracco Italiano [44], and Kooiker [45]. Meanwhile, much higher inbreeding levels were reported for Boxer dogs [30] and Bolognese dog breeds [46]. According to Pekkala et al. [47], inbreeding and genetic drift increase in small or closed populations since there are fewer breeding individuals contributing to each generation. The high levels of inbreeding in sizable Boxer [30] and Bolognese dog breeds [46] may be due to a reduced effective population. Wijnrocx et al. [48] found a low number of founders (3) in a re-established Bouvier des Ardennes dog population, which led to a higher *F* (44.7), indicating a loss of genetic diversity. The observed rate of inbreeding per year (0.20%) was within the acceptable range of 0.5% per year or 0.5 to 1% according to the Food and Agriculture Organization [31]. Similarly, the effective population size observed in the BBD (83.1) was within the recommended range of 50–100 animals, allowing for a 1% increase in the inbreeding rate per generation. Meanwhile, Meuwissen and Woolliams [49] suggested an *Ne* value of 31–250 for maintaining population fitness. Even though the *Ne* value in the BBD is clear of critical or dangerous rates, it is recommended for management to promote the improvement of genetic diversity by ensuring an equal ratio of males and females in mating programs [50] and adding more genetically distant individuals.

Continuous selection for different breeding objectives, such as for exhibitions, shows, and service, may possibly lead to the extinction of a dog breed [39], as intense selection of families increases the risk of undesirable recessive alleles that threaten population fitness and adaptability. Therefore, evaluating the genetic structure of the BBD population is important for the establishment and maintenance of its genetic diversity while managing inbreeding levels to ensure that they do not affect future selection [51]. With sufficient pedigrees, breeding strategies can be planned to prevent related animals from mating [52]. In so doing, Ocampo et al. [53] suggested introducing a higher number of breeding males in each family group to avoid inbreeding and maintain the genetic diversity of the population.

It was observed that male and female Boerboel dogs contributed similarly to the genetic diversity of the breed. According to Fernandes et al. [52], males often contribute more genes to the population, since the male-to-female breeding ratio is greater than one. This implies that a male is used to mate with a large number of females, which is similar to the polygynous mating system described for wild boars (Sus scrofa) [54]. The findings of this study, however, indicate that the numbers of breeding males and females were equal over generations. According to Barros et al. [55], when the proportion of males and females in a population is unequal, the number of animals available for reproduction decreases, which is one of the main causes of genetic diversity loss. As a result, both sexes should transfer significant amounts of the genes that they have in order to maintain diversity [54].

Only ten animals explained 50% of the total genetic variation, implying that major ancestors did not equally contribute to the genetic diversity of the BBD population. This could be due to selection pressure, with some animals or families being preferred more than others [56]. Similar findings were reported for Dogue de Bordeaux dogs [46] and Bracco Italiano dogs [44], but these were lower than those of the ancestors of Deutsch Drahthaar dogs [57]. In this study, male ancestors contributed 10.67% to the total genetic variation, which was similar to the findings of Brito et al. [58] and Sölkner et al. [59] for Austrian Braunvieh, Pinzgauer, and Grauvier cattle populations, where an influential ancestor contributed close to 10% to the total genetic variation. As a result, higher inbreeding levels are associated with low numbers of ancestors explaining high levels of genetic variability in a population [60]. A reasonable strategy for achieving maximum long-term genetic diversity according to Navas et al. [40] is to divide the population into as many different groups as possible. Keeping populations separated decreases the risk of extinction due to accidents or health-related problems, as these events would only affect a single group. However, breeders are unable to maintain the same variability in newly formed subpopulations as in the founder population, despite international efforts to share genetic material [40]. As a result, heterozygosity decreases with each passing generation, while inbreeding increases [61,62].

For founders to contribute equally to the population’s total genetic variation, the ratio of effective founders with respect to the total number of founders ($f_{e}$/*f*) must be equal to one [35]. This ratio indicates whether preferential breeding is taking place [39], and in our study, this ratio was less than unity, with the RP being defined as males (0.16) or females (0.17). Similar ratios were shared by Shariflou et al. [34] for the Australian Cattle Dog and Border Collie, indicating a loss of genetic diversity due to the use of the same animals as parents or unequal contributions of ancestor genes in the population [11,25]. In a population that has not experienced a greater reduction in genetic diversity due to factors such as bottlenecks and genetic drift [25,63], the ratio of effective founders to ancestors ($f_{e}$/$f_{a}$) should be close to one [25,64]. This ratio was greater than unity at 2.1 and 2.0 for males and females, respectively, in our study, indicating the established loss of genetic diversity. These findings were similar to those found for Hannoverian hounds [65], Bichon frise [34], Australian shepherds, and the Papillon and Phalene [48] dog breeds. However, greater bottlenecks were found for Labrador Retriever [66] and Border Collie [67] dogs, respectively, with much higher $f_{e}$/$f_{a}$ ratios of 3.67 and 7.3. The ratio in the BBD of South Africa could also be explained by the population’s closed herd book, strong selection for appearance, and favoritism of a few individuals [39]. According to Bannasch et al. [68], population bottlenecks caused by past events and the creation of closed studbooks in the last hundred years have affected the creation of modern dog breeds. Population bottlenecks can occur due to changes in population size, the geographical isolation of the parent stock, selection pressures, and a change in breeding objectives [56,69,70]. Conservation strategies are required to monitor genetic diversity and inbreeding, e.g., with new selection programs for increasing the gene pool by mating males that have not been mated before [71] or genetically distant dog breeds.

Our research established that genetic diversity in the BBD is low but not eroded. This means the BBD breed is not at risk of extinction. The BBD may possess unique genetic variants that survive into the future. Thus, breeding programs need to maintain this diversity for the continued survival of the BBD and to assist in restoring the diversity of commercial breeds that are likely to lose their diversity through intense selection. Diversity management strategies could identify a reasonable number of animals of genetic value that need to be retained, with sufficient females underlying the population growth rate [61]. Furthermore, management strategies need to ensure equal genetic contributions of parents to future generations to maximize the observed total genetic variation that is explained by a handful of animals. Hierarchical designs and optimal contribution strategies have been explored as a way to balance parental contributions to the population [61].

Pedigree analyses are useful resources for providing a better understanding of populations’ history, and they allow for the identification of historical events, such as founder animals and genetic bottlenecks [72]. Although pedigree data are important for determining genetic diversity, they are limited by the start of recording and missing or incorrect recordings [20]. The availability of genomic data has facilitated landscape genomics and conservation studies to accurately estimate genetic diversity and inbreeding [8,21,22]. Genomic data enable the accurate assessment of a population structure based on the actual versus expected genetic diversity [23]. Genomic information, on the other hand, is still limited by the cost of marker genotyping.

## 5. Conclusions

The BBD population has low genetic diversity, with almost all animals being inbred. We observed uneven genetic contributions of the most important ancestors to the reference population, with only a handful of ancestors contributing more than 50% of the total genetic variation. The rate of increase per year of 0.20% fell at the borderline for ensuring sufficient genetic variability, while the effective population size of 83.1 was clear of critical variability levels. Nevertheless, it is recommended that breeding strategies closely monitor inbreeding rates to maintain or improve the genetic diversity in the BBD. Our study explored genetic diversity using pedigree data, but the accurate estimation of genetic diversity parameters can be achieved using more advanced genomic technologies.

## Figures and Tables

**Table 1:** The inbreeding classes of inbred animals in the pedigree.

Inbreeding Class	No. of Individuals	Proportion of Animals%
0–5	28,664	35.8
5–10	34,668	43.3
10–15	9538	11.9
15–20	4396	5.5
20–25	1225	1.5
25–30	1067	1.3
30–35	340	0.4
35–40	100	0.1
40–45	15	0.02
50–55	1	0
Number of individuals: 87,755		
Number of inbred individuals: 80,014		
Mean inbreeding of inbred individuals: 7.5		
Maximum inbreeding: 50		

**Table 2:** The estimated measures of genetic diversity.

Parameters	Males	Females
Total no. of founders (f)	348	356
Effective no. of founders ($f_{e}$)	57.4	60.1
Effective no. of ancestors ($f_{a}$)	27.93	29.5
$f_{e}/$ *f*	0.16	0.17
$f_{e}/f_{a}$	2.1	2.0
Effective population size (Ne)		83.1

## Data Availability

The availability of these data is restricted. The data were extracted from the Integrated Registration and Genetic Information System (INTERGIS) and are accessible from the authors upon reasonable request with the permission of INTERGIS.
